# Effectiveness of mobile-based monitoring system (ONKOSIS) in the management of chemotherapy-related symptoms: a randomized controlled trial

**DOI:** 10.1007/s00520-026-10512-5

**Published:** 2026-03-04

**Authors:** Unal Onsuz, Gulbeyaz Can

**Affiliations:** 1 Istanbul University-Cerrahpasa Institute of Graduate Studies, Istanbul, Turkey; 2 Kocaeli University Faculty of Health Sciences, Kocaeli, Turkey; 3 Istanbul University-Cerrahpasa Florence Nightingale Nursing Faculty, Istanbul, Turkey

**Keywords:** Cancer, Chemotherapy, MHealth, Symptom management, Quality of life

## Abstract

**Purpose:**

This study was conducted to evaluate the impact of the ONKOSIS mobile application, developed within the scope of the study, on the management of chemotherapy-related symptoms and quality of life.

**Methods:**

This randomized controlled, single-blind experimental design study was carried out with the participation of 199 patients at the Kocaeli University Semahat Aracı Oncology and Palliative Care Center, Outpatient Chemotherapy Unit. In the study, which used simple randomization, the intervention group consisted of 98 patients and the control group consisted of 101 patients. During chemotherapy treatment, in addition to standard care for symptom management, the intervention group used the ONKOSIS mobile application, while the control group used a demo mobile application. The study data were obtained through the Diagnostic Form, Nightingale Symptom Assessment Scale, Coping with Chemotherapy Symptoms Form, and patient satisfaction survey. The follow-up frequency was planned according to the type of treatment protocol applied to the patients. Patients who received four cycles of treatment were followed up after the 2nd, 3rd, and 4th cycles, while patients who received six cycles of treatment were followed up after the 2nd, 4th, and 6th cycles.

**Results:**

The study group had a mean age of 47.18 ± 13.83; 74.4% were female. Treatment cycles of 2, 3, or 4 weeks were provided for 26.6% of patients receiving treatment for gastrointestinal cancer, 21.6% for breast cancer, and 17.1% for lung cancer. Sociodemographic and clinical characteristics were similar between groups. Although the intervention group exhibited greater initial symptom burden (higher N-SAS scores at T1/T2), their symptom trajectory improved significantly over time compared to the control group, supporting the role of mHealth in enhancing outcomes. The use of ONKOSIS improved quality of life and patient satisfaction but did not reduce unscheduled healthcare visits.

**Conclusion:**

The ONKOSIS mobile application was found to be an effective tool in supporting symptom management and improving the quality of life for cancer patients during the treatment process. It was determined that the widespread adoption of the mobile application in cancer care could contribute to improving patient outcomes and enhancing care quality.

**Trial registration:**

ClinicalTrials.gov ID: NCT05946070. Registered on June 22, 2023

## Introduction

Cancer is a significant global public health problem, representing the second leading cause of mortality after heart disease. This disease, which is characterized by a wide spectrum of approximately 200 distinct types, is the result of a process involving genetic mutations, with an increasing annual number of cases being reported [1, 2]. In 2022, the number of new cancer cases was approximately 20 million, and it is projected that this figure will reach 32.6 million by 2045. In Turkey, 240,013 new cases were reported in 2022, and it is estimated that this number will rise to 419,201 by 2045 [3]. Despite the advances in early diagnosis and treatment techniques, the side effects of cancer treatments have a detrimental effect on patients’ quality of life [4]. Chemotherapy targets rapidly dividing cells, but also damages healthy cells, causing side effects such as fatigue, nausea, vomiting, anxiety, and pain [5]. These side effects have been shown to impact individuals’ physical, psychosocial, and functional well-being, thereby hindering adherence to treatment [6, 7]. The development of skills in the management of symptoms and self-management is a factor in the improvement of treatment outcomes and the facilitation of a more expeditious return to daily life for patients undergoing chemotherapy [8].

In recent years, digital health technologies have emerged as a promising area in cancer care. Mobile health applications (mHealth) have been demonstrated to be an effective tool for increasing patient engagement, facilitating symptom tracking, and improving access to healthcare services [9]. Digital solutions provided through mobile devices have been demonstrated to enable patients to report their symptoms and receive personalized treatment recommendations [10, 11]. The utilization of electronic health technologies to facilitate patient-reported outcomes (ePRO) has been demonstrated in managing symptoms and the decision-making processes related to health. Randomized controlled trials have demonstrated that electronic ePRO systems enhance quality of life and reduce the frequency of emergency department visits and hospitalization rates [12]. Furthermore, these technologies empower patients to proactively engage in their own care processes, thereby enhancing their self-management skills [13].

The mobile application developed within the scope of the study was designated the Oncology Symptom Management System (ONKOSIS), as it reflects the concepts related to the research. In this study, a randomized controlled trial was conducted to evaluate the impact of the ONKOSIS mobile application on the management of symptoms and the quality of life of cancer patients undergoing chemotherapy. The study tested the hypotheses that the ONKOSIS application would improve the quality of life of patients in the intervention group (H1), would be more successful in managing chemotherapy-related symptoms (H2), would reduce the frequency of visits to healthcare facilities due to side effects (H3), and would increase patient satisfaction (H4). The research results are expected to highlight the potential of digital health technologies to increase patient participation in cancer treatment and improve access to healthcare services. In this context, the study aims to make an important contribution to the effectiveness of personalized digital solutions in cancer treatment.

## Method

### The development process of the ONKOSIS mobile application

The Analysis, Design, Development, Implementation, and Evaluation (ADDIE) model, a widely utilized framework within the domain of educational technology, served as the foundational methodology for the process of developing the ONKOSIS mobile application [14]. In the course of the analysis phase, the requirements of the target demographic (cancer patients), with regard to domestic management of symptoms, were identified, and user profiles and content consistent with current literature were created. During the design phase, the user experience and visual design of the application incorporated elements such as a user-friendly interface to ensure fast and efficient operation, a modern appearance, secure data storage, and compatibility. During the development phase, the application was coded, tested, and published using React Native and Firebase technologies, with the latter being used for content and survey management. The ONKOSIS mobile application was subsequently made available on the Google Play Store and promoted in the application phase. During the evaluation phase, the application’s effectiveness and user satisfaction were assessed through user experience testing and feedback. Following the findings, improvements were initiated. The application’s content validity was evaluated through Davis’s Content Validity Index and expert opinions. The result of the calculation of the Content Validity Index was 0.98, which indicates that the content is highly valid [15]. The readability of the content was evaluated using the Ateşman Readability Index, which determined it to be in the “easy” category with a score of 70.2 [16]. As part of the preliminary research application, a pilot study was conducted with ten patients who met the research criteria, at the Semahat Aras Oncology and Palliative Care Centre Outpatient Chemotherapy Unit at Kocaeli University. A survey was conducted to solicit user feedback regarding the application, and the content was subsequently revised to address issues pertaining to spelling, technical glitches, and readability. The development process of the ONKOSIS mobile application was then carried out systematically within the framework of the ADDIE model, thereby increasing the application’s effectiveness with user-focused improvements [17]. The ONKOSIS architecture was strategically designed to establish a direct functional link between the intervention’s features and the study’s clinical endpoints. In contrast to passive educational tools, the application employed active decision-support algorithms that provided real-time, symptom-specific guidance, thereby directly facilitating the primary outcome of patient participation in symptom management. To address the complex challenge of unplanned healthcare utilization, the system was equipped with a rapid-response communication module. By enabling researchers to evaluate symptoms and reply to patient queries within 30 to 60 min—7 days a week—this feature functioned as an effective remote triage mechanism, preventing unnecessary emergency visits for conditions that could be managed at home. Meanwhile, patient satisfaction was primarily driven by the integration of prompt professional responsiveness and the streamlined, validated user interface.

### Study design and sample

This single-center, single-blind, randomized controlled trial was conducted at the Semahat Aracı Oncology and Palliative Care Centre, Kocaeli University, from July 2023 to May 2024. Eligible participants were cancer patients aged 18 or older, scheduled for their first chemotherapy, and owning an Android smartphone. Inclusion criteria included the completion of at least four chemotherapy cycles, a treatment plan with 2–4-week intervals, and the ability to understand and use the mobile application. Exclusion criteria included physical or cognitive impairments hindering app use, concurrent radiotherapy, and communication barriers. Participants were also excluded upon withdrawal, health deterioration, treatment discontinuation, protocol changes, transfer to another institution, or death.

The initial sample size was determined using G*Power 3.1.9.2 software based on a repeated-measures (within-between interaction) design, with the N-SAS scores as the primary outcome. To detect a medium effect size (Cohen’s *f* = 0.25 or *d* = 0.5) with a significance level (*α*) of 0.05 and a statistical power of 0.80, a minimum of 196 participants was required. To account for an anticipated attrition rate of approximately 10%, 212 patients were initially enrolled. The study was completed with 199 participants (intervention, *n* = 98; control, *n* = 101), resulting in a final attrition rate of 6.13%. A post hoc power analysis conducted using the actual study data revealed an observed effect size of *d* = 2.27, confirming that the final sample size provided robust statistical power to detect significant differences.

The study was approved by the Istanbul University-Cerrahpaşa Social and Human Sciences Research Ethics Committee (decision no. 2020-192), and written informed consent was obtained. The Standard Protocol Items: Recommendations for Interventional Trials (SPIRIT) 2013 checklist and Consolidated Standards of Reporting Trials (CONSORT) 2010 flow diagram guided study planning. The study protocol was registered on ClinicalTrials.gov (Identifier: NCT05946070; registration date: June 22, 2023) prior to the enrollment of the first participant.

### Randomization and blinding

Simple randomization was used to allocate 199 patients to intervention (*n* = 98) and control (*n* = 101) groups. Randomization, using a random number table, followed the order in which eligible patients consulted the chemotherapy education nurse. Participants were assigned sequentially to ensure balanced group distribution. To minimize contamination bias, interaction between groups was limited. The process was designed to maximize group similarity and study validity. While researchers could not be blinded due to the intervention’s nature, patients remained blinded to group allocation.

### Intervention and control group

Patients in the intervention group used the ONKOSIS mobile application alongside standard care. ONKOSIS is a specialized app developed to monitor and manage chemotherapy-related symptoms, enabling patients to report symptoms after each treatment cycle and receive personalized recommendations based on international evidence-based guidelines and expert opinions [18–21]. The app includes algorithms for 28 symptoms, providing automatic self-care advice according to symptom severity, and it features an instant messaging module. Before use, participants received comprehensive training, including demonstrations, documents, and videos, to ensure familiarity with the app. The installation process was assisted by a technician, and the initial symptom entries were completed together with the user. Patients completed the Nightingale Symptom Assessment Scale (N-SAS) via the app every 14 days. Reminders were sent to ensure timely reporting; if reports were not submitted within two days, additional reminders followed. By the third day, researchers contacted patients to address any issues. Symptom data were provided to patients in graphical and written formats. The app did not include an alert system, but researchers could monitor symptom severity via a web panel. Control group patients used a demonstration application that only collected data and did not provide symptom management content, though it included instant messaging. They received standard educational materials and were instructed to use the demo app for symptom monitoring. Data collection included the Diagnostic Form (sociodemographic and clinical characteristics), N-SAS (symptom severity), Chemotherapy Symptom Management Form (intervention efficacy), and Patient Satisfaction Survey (opinions and satisfaction with ONKOSIS). All tools were completed during the study and used to test research hypotheses.

### Outcome measures

#### Outcome measures and conceptual framework

The study outcomes were categorized into primary outcomes, secondary outcomes, and confounding factors, with each construct operationalised as follows:

#### Primary outcome

##### Symptom burden and quality of life

The primary construct evaluated was symptom burden and its impact on quality of life. This was assessed using the N-SAS. Conceptually, N-SAS evaluates the severity of symptoms developed due to cancer treatment and their interference with daily life across three specific domains: physical well-being (e.g., pain, nausea), social well-being (e.g., interaction difficulties), and psychological well-being (e.g., anxiety, distress). Higher scores on this 38-item Likert-type scale indicate greater symptom severity and poorer quality of life [22]. The scale demonstrated high internal consistency in this study (Cronbach’s alpha, T1 = 0.975, T2 = 0.941, T3 = 0.887).

#### Secondary outcomes

##### Unplanned healthcare utilization

This outcome represents the system-level impact of symptom management. It was operationalized as the frequency of unscheduled medical interactions required due to chemotherapy side effects. Data were collected on emergency department visits, unscheduled outpatient visits, and hospital admissions, serving as a proxy for the efficacy of the self-management intervention in preventing acute care needs. To capture the full extent of utilization, this outcome was calculated cumulatively across the entire follow-up period (T1–T3). Given that hospital visits are discrete events, the variable was operationalized in two formats for analysis: (1) as a categorical variable based on frequency (0, 1, 2, and ≥ 3 visits) and (2) as a binary outcome (presence vs. absence of any unscheduled visit).

##### Patient satisfaction

This construct assessed the patients’ subjective perception of support efficacy. It was measured using a Visual Analog Scale (0–10). For the intervention group, this reflected satisfaction with the mobile application’s guidance; for the control group, it reflected satisfaction with standard care.

##### Covariates and confounding factors

Sociodemographic characteristics (age, education, marital status) and clinical variables (diagnosis, treatment protocol, stage), which could influence symptom perception and app usage, were collected via the Diagnostic Form at baseline to be controlled for in the analysis.

### Statistical analysis

Data were analyzed using IBM SPSS Statistics 21.0. Continuous variables were expressed as mean ± standard deviation or median (IQR), and categorical variables as numbers and percentages. Internal consistency of the N-SAS was evaluated using Cronbach’s alpha. Normality was assessed via the Kolmogorov–Smirnov test, skewness/kurtosis values, and box plots. Group comparisons were performed using Student’s *t*-test for normally distributed variables and Mann–Whitney *U* test for non-normal distributions. Pearson’s and Yates’ chi-square tests were used for categorical data. Temporal changes in non-normally distributed data were evaluated with the Friedman test and Bonferroni–Dunn post hoc analysis. To assess the longitudinal effect of the intervention and potential moderating effects of patient characteristics (e.g., age, gender, tumor type), three-way mixed-design ANOVA was conducted. Time (T1–T3) served as the within-subjects factor, while group and demographic/clinical variables were between-subjects factors. Sphericity was checked via Mauchly’s test, applying Greenhouse–Geisser corrections where necessary. Due to the zero-inflated and right-skewed nature of unplanned healthcare utilization data, Pearson’s chi-square test was used for proportional group comparisons. Statistical significance was set at *p* < 0.05. 

## Results

**Fig. 1:**
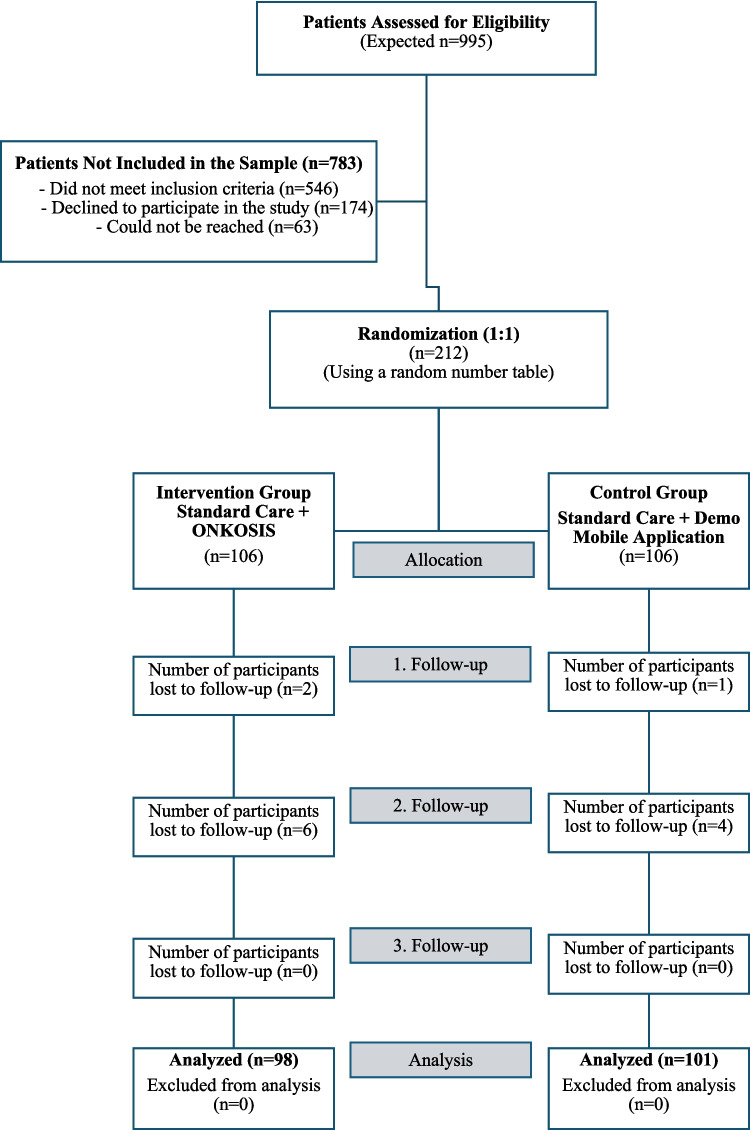
CONSORT 2010 flow diagram

A total of 199 patients were included in the study, with 98 in the experimental group and 101 in the control group (Fig. 1). The mean age of the participants was 47.18 ± 13.83 years; 58.3% were female and 41.7% male. The most common tumor types were gastrointestinal (26.6%), breast (21.6%), and lung (17.1%) cancers. Statistical analysis confirmed that randomization resulted in a homogeneous distribution between the groups in terms of demographic and clinical characteristics. As shown in Table 1, no significant differences were observed between the groups regarding age, gender, educational status, treatment type, or treatment frequency (*p* > 0.05). To further evaluate the effectiveness of the intervention and investigate potential moderating effects of patient characteristics on symptom management, a series of three-way mixed ANOVAs was performed. These analyses tested the three-way interactions between time (T1, T2, T3), group assignment (intervention vs. control), and various demographic/clinical factors. The results revealed no statistically significant three-way interactions for age (*p* = 0.293), gender (*p* = 0.203), educational status (*p* > 0.05), tumor type (*p* > 0.05), or treatment protocol (*p* > 0.05) regarding N-SAS total scores. These findings indicate that the trajectory of symptom burden reduction, as measured by the N-SAS, remained consistent across all subgroups. This suggests that the clinical efficacy of the ONKOSIS intervention is maintained across diverse patient profiles and is independent of the evaluated demographic and clinical variables.

**Table 1:** The distribution of basic characteristics

Features	Total (*n* = 199)	Experimental group (*n* = 98)	Control group (*n* = 101)	Statistics	*p*
	*n* (%)	*n* (%)	*n* (%)		
Province				0.131^a^	0.717
Kocaeli Provinces other than Kocaeli	148 (74.4) 51 (25.6)	74 (75.5) 24 (24.5)	74 (73.3) 27 (26.7)		
Gender				0.063^a^	0.801
Female Male	116 (58.3) 83 (41.7)	58 (59.2) 40 (40.8)	58 (57.4) 43 (42.6)		
Marital status				2.961^b^	0.085
Married Single/widowed	162 (81.4) 37 (18.6)	85 (86.7) 13 (13.3)	77 (76.2) 24 (23.8)		
Educational status				4.657^a^	0.199
Primary education Middle school High school Undergraduate and graduate	83 (41.7) 23 (11.6) 48 (24.1) 45 (22.6)	47 (48.0) 12 (12.2) 18 (18.4) 21 (21.4)	36 (35.6) 11 (10.9) 30 (29.7) 24 (23.8)		
Occupation				5.785^a^	0.216
Civil servant Worker Retired Housewife Other	25 (12.6) 12 (6.0) 43 (21.6) 76 (38.2) 43 (21.6)	13 (13.3) 7 (7.1) 20 (20.4) 43 (43.9) 15 (15.3)	12 (11.9) 5 (5.0) 23 (22.8) 33 (32.7) 28 (27.7)		
Tumor type				3.432^a^	0.488
Lung Breast GI Gynecological Other	34 (17.1) 43 (21.6) 53 (26.6) 20 (10.1) 49 (24.6)	16 (16.3) 19 (19.4) 23 (23.5) 11 (11.2) 29 (29.6)	18 (17.8) 24 (23.8) 30 (29.7) 9 (8.9) 20 (19.8)		
Type of treatment				0.656^b^	0.418
Adjuvant Neoadjuvant	176 (88.4) 23 (116)	89 (90.8) 9 (9.2)	87 (86.1) 14 (13.9)		
Use of immunotherapy/targeted therapy				0.201^a^	0.654
Yes No	58 (29.1) 141 (70.9)	30 (30.6) 68 (69.4)	28 (27.7) 73 (72.3)		
Treatment Interval				3.369^a^	0.186
2-week interval 3-week interval 4-week interval	87 (43.7) 68 (34.2) 44 (22.1)	37 (37.8) 39 (39.8) 22 (22.4)	50 (49.5) 29 (28.7) 22 (21.8)		
Age (year)				−0.802^c^	0.424
Mean ± SD (Min–Max)	47.18 ± 13.83 (18–65)	47.98 ± 13.44 (19–65)	46.41 ± 14.22 (18–65)		

Values are presented as *n* (%) or mean ± SD (minimum–maximum)

ᵃPearson’s chi-square test

ᵇYates’ continuity-corrected chi-square test

ᶜIndependent samples Student’s *t*-test

In the evaluation conducted with N-SAS, a decrease was observed in the percentages of respondents in the experimental group who answered “very much” and “quite a lot” in the physical, social, and psychological well-being sub-dimensions from T1 to T3, while these percentages generally increased or remained stable in the control group. The proportion of responses in the “somewhat” and “very little” categories also decreased over time in the experimental group, while these proportions generally increased or remained unchanged in the control group.

**Fig. 2:**
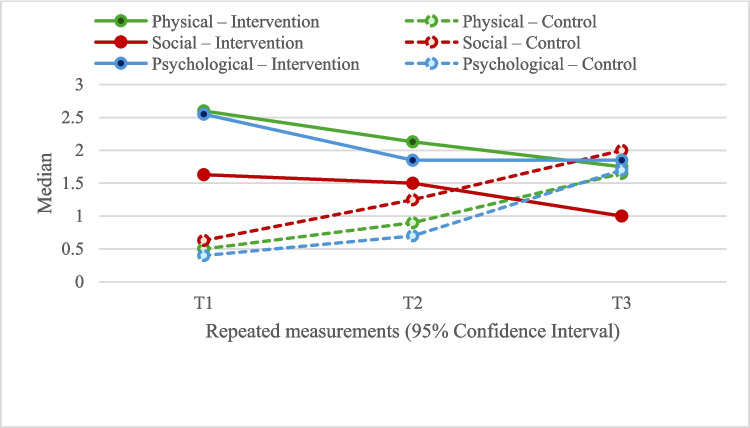
Changes in physical, social, and psychological well-being scores

When physical well-being scores were examined, the experimental group scored significantly higher than the control group in both T1 and T2 measurements (T1, 2.60 [0.81] vs. 0.50 [0.70], *p* < 0.001; T2, 2.13 [0.80] vs. 0.90 [0.93], *p* < 0.001). However, no significant difference was found between the groups at T3 (T3, 1.75 [0.50] vs. 1.65 [1.00], *p* = 0.082). In the experimental group, a significant decrease in physical well-being scores was observed from T1 to T3 (T2–T1, −0.40 [0.30], *p* < 0.001; T3–T1, −0.70 [0.41], *p* < 0.001; T3–T2, −0.35 [0.90], *p* < 0.001), whereas an increase in scores was observed in the control group (T2–T1, 0.40 [0.30], *p* < 0.001; T3–T1, 1.15 [0.48], *p* < 0.001; T3–T2, 0.70 [0.20], *p* < 0.001) (Fig. 2) (Table 2).

**Table 2:** Comparison of N-SAS well-being subscale scores

Subscales of N-SAS	Measurements	Intervention group (***n*** = 98)	Control group (***n*** = 101)	Test statistic	***p***
**Physical well-being**	T1 T2 T3 Test statistic/*p*	2.60 (0.81) 2.13 (0.80) 1.75 (0.50) **193.140/0.000**^b^	0.50 (0.70) 0.90 (0.93) 1.65 (1.00) **202.000/0.000**^b^	−12.136 −10.323 −1.742	**0.000**ᵃ **0.000**ᵃ 0.082ᵃ
	**Change ∆**				
	T2–T1 Test statistic/*p*	−0.40 (0.30) **7.214/0.000**^bb^	0.40 (0.30) **−7.106/0.000**^bb^	−12.195	**0.000ᵃ**
	T3–T1 Test statistic/*p*	−0.70 (0.41) **13.786/0.000**^bb^	1.15 (0.48) **−14.213/0.000**^bb^	−12.190	**0.000**ᵃ
	T3–T2 Test statistic/*p*	−0.35 (0.90) **6.571/0.000**^bb^	0.70 (0.20) **−7.106/0.000**^bb^	−12.203	**0.000**ᵃ
**Social well-being**	T1 T2 T3 Test statistic/*p*	1.63 (1.13) 1.50 (0.78) 1.00 (0.50) **127.074/0.000**^b^	0.63 (0.88) 1.25 (1.13) 2.00 (1.19) **202.000/0.000**^b^	−8.163 −2.685 −9.313	**0.000**ᵃ **0.007**ᵃ **0.000**ᵃ
	**Change ∆**				
	T2–T1 Test statistic/*p*	−0.13 (0.38) 1.571/0.348^bb^	0.63 (0.38) **−7.106/0.000**^bb^	−12.243	**0.000ᵃ**
	T3–T1 Test statistic/*p*	−0.50 (0.66) **10.107/0.000**^bb^	1.38 (0.38) **−14.214/0.000**^bb^	−12.212	**0.000**ᵃ
	T3–T2 Test statistic/*p*	−0.38 (0.38) **8.536/0.000**^bb^	0.75 (0.25) **−7.106/0.000**^bb^	−12.269	**0.000**ᵃ
**Psychological well-being**	T1 T2 T3 Test statistic/*p*	2.55 (0.90) 1.85 (0.93) 1.85 (0.53) **139.877/0.000**^b^	0.40 (0.70) 0.70 (0.85) 1.70 (1.10) **196.148/0.000**^b^	−12.057 −9.534 −2.553	**0.000**ᵃ **0.000**ᵃ **0.011**ᵃ
	**Change ∆**				
	T2–T1 Test statistic/*p*	−0.55 (0.30) **10.036/0.000**^bb^	0.30 (0.40) **−6.192/0.000**^bb^	−12.212	**0.000ᵃ**
	T3–T1 Test statistic/*p*	−0.60 (0.60) **10.000/0.000**^bb^	1.30 (0.60) **−13.755/0.000**^bb^	−12.197	**0.000**ᵃ
	T3–T2 Test statistic/*p*	0.00 (0.40) −0.036/1.000^bb^	0.90 (0.40) **−7.564/0.000**^bb^	−12.214	**0.000**ᵃ

Values are presented as median (interquartile range). Values in bold indicate statistical significance.

ᵃMann–Whitney *U* test

ᵇFriedman test

ᵇᵇDunn test with Bonferroni correction

In terms of social well-being scores, the experimental group demonstrated significantly higher scores in T1 and T2 (T1, 1.63 [1.13] vs. 0.63 [0.88], *p* < 0.001; T2, 1.50 [0.78] vs. 1.25 [1.13], *p* = 0.007). Conversely, at T3, the control group exhibited significantly higher scores in comparison to the experimental group (T3, 1.00 [0.50] vs. 2.00 [1.19], *p* < 0.001). In the experimental group, a significant decrease in social well-being scores was observed from T1 to T3 (T2–T1, −0.13 [0.38], *p* = 0.348; T3–T1, −0.50 [0.66], *p* < 0.001; T3–T2, −0.38 [0.38], *p* < 0.001), while there was observed an increase in the control group (T2–T1, 0.63 [0.38], *p* < 0.001; T3–T1, 1.38 [0.38], *p* < 0.001; T3–T2, 0.75 [0.25], *p* < 0.001) (Fig. 2) (Table 2).

With regard to psychological well-being scores, the experimental group demonstrated significantly higher scores than the control group at T1, T2, and T3 measurements (T1, 2.55 [0.90] vs. 0.40 [0.70], *p* < 0.001; T2, 1.85 [0.93] vs. 0.70 [0.85], *p* < 0.001; T3, 1.85 [0.53] vs. 1.70 [1.10], *p* = 0.011). In the experimental group, a significant decrease in psychological well-being scores was observed from T1 to T3 (T2–T1, −0.55 [0.30], *p* < 0.001; T3–T1, −0.60 [0.60], *p* < 0.001; T3–T2, 0). A statistically significant increase was observed in the control group (T2–T1, 0.30 [0.40], *p* < 0.001; T3–T1, 1.30 [0.60], *p* < 0.001; T3–T2, 0.90 [0.40], *p* < 0.001) (Fig. 2) (Table 2).

**Fig. 3:**
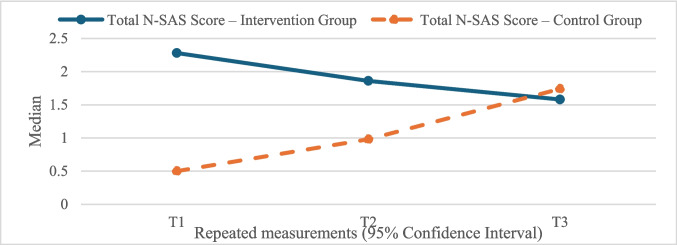
Change in total N-SAS score by group

The total N-SAS scores of the experimental group were significantly higher than those of the control group in the T1 and T2 measurements (T1, −11.912; *p* < 0.001; T2, −8.386; *p* < 0.001). However, at T3, the control group’s scores were significantly higher than those of the experimental group (T3, −2.163; *p* = 0.031). There was a significant decrease in total scores from T1 to T3 in the experimental group (T3–T1, 13.429; *p* < 0.001), and a significant increase in the control group (T3–T1, −14.213; *p* < 0.001). Temporal changes were statistically significant in both groups. These results suggest that the ONKOSIS application initially had a positive impact on the well-being of the experimental group, but this effect diminished over time, whereas well-being scores in the control group increased (Fig. 3).

When comparing the groups in terms of their ability to cope with symptoms, satisfaction, and the occurrence of out-of-follow-up visits, it was found that the entire experimental group stated that ONKOSIS was effective in coping with symptoms, while the entire control group stated that the demo application was ineffective (*p* < 0.001). No statistically significant differences were observed between the groups with respect to out-of-follow-up visit rates and intervention rates during visits (*p* > 0.05). Furthermore, the satisfaction level of the experimental group with the mobile health application was significantly higher than that of the control group (*p* < 0.001) (Table 3).

**Table 3:** Comparison of the characteristics of coping, satisfaction, and non-protocol visits between the groups

Characteristics	Total (*n* = 199)	Intervention group (*n* = 98)	Control group (*n* = 101)	Test statistic	*p*
	*n* (%)	*n* (%)	*n* (%)		
Coping with symptoms Effective Ineffective	98 (49.2) 101 (50.8)	98 (100.0) 0 (0.0)	0 (0.0) 101 (100.0)	199.00^a^	**0.000**
Unscheduled visits 0 1 2 3 and more	98 (49.2) 47 (23.6) 25 (12.6) 29 (14.6)	50 (51.0) 23 (23.5) 12 (12.2) 13 (13.3)	48 (47.5) 24 (23.8) 13 (12.9) 16 (15.8)	0.367^a^	0.947
Intervention during unscheduled visit Yes No	53 (26.6) 146 (73.4)	23 (23.5) 75 (76.5)	30 (29.7) 71 (70.3)	0.989^a^	0.320
Satisfaction Med. (IQR)	9.00 (0.00)	9.00 (0.00)	9.00 (2.00)	−6.136^b^	**0.000**

Values are presented as *n* (%) or median (interquartile range). Values in bold indicate statistical significance

ᵃPearson’s chi-square test

ᵇMann–Whitney *U* test

## Discussion

A significant finding of this randomized controlled trial is that the clinical benefit provided by the ONKOSIS intervention remained consistent, regardless of the patients’ demographic backgrounds or clinical diagnostic characteristics. Specifically, the lack of significant differences in N-SAS reporting processes among older patients or those with varying educational levels suggests that the “technology use barrier,” frequently encountered in digital health applications, was successfully addressed in this study. The stable trajectory of symptom management success across various subgroups indicates that N-SAS reporting via the ONKOSIS system was effectively performed regardless of patient profiles.

Contrary to some existing literature, these results provide evidence that with appropriate education and support, mobile applications can be effective across different patient groups [23–26]. Similarly, our findings align with studies demonstrating that age and educational level are not consistently significant determinants of adherence to cancer-related mHealth applications [27]. The fact that the clinical impact of the intervention was maintained independently of sociodemographic variables supports the view that potential limitations related to age or educational level in reporting symptoms via mHealth tools were kept to a minimum within the scope of this study.

The findings indicate that patients in the ONKOSIS intervention group had significantly higher N-SAS total and subscale scores (physical, social, and psychological well-being) at early treatment stages (T1 and T2), reflecting greater baseline and early symptom burden compared to the control group. However, over time, the intervention group demonstrated a downward trajectory in N-SAS scores, indicating a significant improvement in symptom severity. In contrast, the control group showed increasing or minimally improved scores across the same period, suggesting worsening or persistent symptom burden during treatment. Overall, although the intervention group began with more severe symptoms, their subsequent improvement trajectory was more favorable than that of the control group. These findings are consistent with prior research suggesting that mHealth-supported symptom self-management and care connectivity may contribute to improved symptom management performance and subjective satisfaction [4, 8, 12, 28–33].

In the present study, while the adherence rate to the application was high, instances of incomplete symptom reporting were noted. Since the study did not formally assess the specific causes of non-compliance or evaluate missing-data mechanisms, no causal inference can be drawn regarding the factors that may have influenced digital engagement. Furthermore, the 14-day reporting interval may have impacted engagement frequency compared to daily prompts. These observations align with extant literature suggesting that shorter reporting intervals, coupled with additional motivational support, may be pivotal in enhancing adherence to digital symptom monitoring systems [12, 34, 35].

The results of the study demonstrated increased engagement in symptom management and improvement in self-management skills among patients who utilized the ONKOSIS application. The application’s user-friendly interface and instant messaging module enabled patients to report their symptoms more effectively and enhanced their communication with the healthcare team. The relevant literature also indicates that mobile applications facilitate real-time symptom monitoring and patient–healthcare provider communication, thereby encouraging more active patient participation in treatment [9–11, 29, 36]. In particular, it has been shown that applications such as electronic Patient-Reported Outcomes version of the Common Terminology Criteria for Adverse Events (PRO-CTCAE) and similar platforms enhance patients’ symptom awareness and enable more open communication with the healthcare team [37, 38]. In our study, we also found that the functionalities of the ONKOSIS application led to increased rates of symptom reporting and higher levels of patient satisfaction.

However, it was noted that the ONKOSIS application did not achieve the expected effect in reducing non-protocol healthcare visits. No significant difference was found between the intervention and control groups in terms of unplanned healthcare facility visits. Similarly, while some studies have reported that mobile health applications reduce emergency department visits and hospitalization rates [12], others have indicated that this effect is limited or not observed [33, 34, 39]. The observed variations in outcomes may be ascribed to a range of factors, including demographic and clinical characteristics of patient populations, the frequency of application use, and the structure of the healthcare system.

The impact of the ONKOSIS application on quality of life was most evident during the early phases of treatment, but diminished over time. This finding, as reported in other studies, suggests that mobile applications may be more effective in the short term, while long-term effectiveness could be enhanced by updating the content of the application and maintaining patient motivation [4, 10, 30, 35]. Moreover, the positive effects of the application on psychological well-being are consistent with findings indicating that mHealth applications may also be beneficial in managing psychological symptoms such as depression, anxiety, and stress [4, 28, 40, 41].

The findings of the present study demonstrate a noteworthy positive effect of the ONKOSIS application on patient satisfaction. Patient satisfaction scores were found to be significantly higher in the intervention arm. The relevant literature also demonstrates that mobile health applications increase patient satisfaction and improve both treatment adherence and self-management skills [28, 32, 39, 40, 42, 43]. The educational materials provided prior to application use and the instant notifications supported more active patient participation in the treatment process.

This study has several limitations. As a single-center study with participants aged 18–65, generalizability may be limited to similar populations. Technically, the requirement for an active internet connection remains a factor in accessibility, despite mitigation via hospital Wi-Fi. Logistical and financial constraints also prevented an Apple Store release; although addressed by using relatives’ Android devices for some participants, the reliance on Android OS introduces potential selection bias. Furthermore, while our analyses suggest that the intervention exhibited a consistent effect across various subgroups, the limited sample size in certain specific clinical categories (e.g., rare tumor types) may have constrained the statistical power for these particular groups. Therefore, a cautious approach is recommended when generalizing the findings to these specific subsets. Finally, the high frequency of zero events in unplanned healthcare utilization data limited the power to detect significant differences in this outcome.

In conclusion, the ONKOSIS mobile application has proven to be an effective digital tool for supporting the management of symptoms, quality of life, and patient satisfaction among cancer patients undergoing chemotherapy. The integration of mobile health applications into cancer care may enhance patient engagement and treatment adherence; however, it is necessary to evaluate their long-term effectiveness and applicability across diverse patient populations. In the future, multicenter and long-term randomized controlled trials are required to assess the impact of mobile health applications on clinical outcomes.

## Data Availability

The data that support the findings of this study are available on request from the corresponding author.

## Code availability

The codes that support the findings of this study are available on request from the corresponding author.

## Abbreviations

*ONKOSIS*: Oncology Symptom Management System
*ePRO*: Patient-reported outcomes
*ADDIE*: Analysis, Design, Development, Implementation, and Evaluation
*SPIRIT*: Standard Protocol Items: Recommendations for Interventional Trials
*CONSORT*: Consolidated Standards of Reporting Trials
*N-SAS*: Nightingale Symptom Assessment Scale
*PRO-CTCAE*: Patient-Reported Outcomes version of the Common Terminology Criteria for Adverse Events
